# Isolation and expression analysis of cDNAs that are associated with alternate bearing in *Olea europaea* L. cv. Ayvalık

**DOI:** 10.1186/1471-2164-14-219

**Published:** 2013-04-03

**Authors:** Ekrem Dündar, Öznur Suakar, Turgay Unver, Ayhan Dagdelen

**Affiliations:** 1 Fen Edebiyat Fakültesi, Biyoloji Bölümü, Balıkesir Üniversitesi, Balıkesir, TURKEY; 2 Fen Fakültesi, Biyoloji Bölümü, Çankırı Karatekin Üniversitesi, Çankırı, TURKEY; 3 Bandırma Meslek Yüksek Okulu, Balıkesir Üniversitesi, Bandırma, TURKEY

**Keywords:** Olive, *Olea europaea* L, Periodicity associated genes, Gene onthology, KEGG

## Abstract

**Background:**

Olive cDNA libraries to isolate candidate genes that can help enlightening the molecular mechanism of periodicity and / or fruit production were constructed and analyzed. For this purpose, cDNA libraries from the leaves of trees in “on year” and in “off year” in July (when fruits start to appear) and in November (harvest time) were constructed. Randomly selected 100 positive clones from each library were analyzed with respect to sequence and size. A fruit-flesh cDNA library was also constructed and characterized to confirm the reliability of each library’s temporal and spatial properties.

**Results:**

Quantitative real-time RT-PCR (qRT-PCR) analyses of the cDNA libraries confirmed cDNA molecules that are associated with different developmental stages (e. g. “on year” leaves in July, “off year” leaves in July, leaves in November) and fruits. Hence, a number of candidate cDNAs associated with “on year” and “off year” were isolated. Comparison of the detected cDNAs to the current EST database of GenBank along with other non - redundant databases of NCBI revealed homologs of previously described genes along with several unknown cDNAs. Of around 500 screened cDNAs, 48 cDNA elements were obtained after eliminating ribosomal RNA sequences. These independent transcripts were analyzed using BLAST searches (cutoff E-value of 1.0E-5) against the KEGG and GenBank nucleotide databases and 37 putative transcripts corresponding to known gene functions were annotated with gene names and Gene Ontology (GO) terms. Transcripts in the biological process were found to be related with metabolic process (27%), cellular process (23%), response to stimulus (17%), localization process (8.5%), multicellular organismal process (6.25%), developmental process (6.25%) and reproduction (4.2%).

**Conclusions:**

A putative P450 monooxigenase expressed fivefold more in the “on year” than that of “off year” leaves in July. Two putative dehydrins expressed significantly more in “on year” leaves than that of “off year” leaves in November. Homologs of UDP – glucose epimerase, acyl - CoA binding protein, triose phosphate isomerase and a putative nuclear core anchor protein were significant in fruits only, while a homolog of an embryo binding protein / small GTPase regulator was detected in “on year” leaves only. One of the two unknown cDNAs was specific to leaves in July while the other was detected in all of the libraries except fruits. KEGG pathway analyses for the obtained sequences correlated with essential metabolisms such as galactose metabolism, amino sugar and nucleotide sugar metabolisms and photosynthesis. Detailed analysis of the results presents candidate cDNAs that can be used to dissect further the genetic basis of fruit production and / or alternate bearing which causes significant economical loss for olive growers.

## Background

Olive (*Olea europaea* L.) has long been among important topics of agricultural research due to its well - known nutritional and health value. Therefore, numerous studies on physiological [1-4] phytochemical [5-9], molecular systematic [10-13] and molecular genetics / genomics [14-17] aspects of olive have been reported. Further genetic studies involving molecular mechanism of fruit set, fruit development, fruit detachment and alternate bearing in olive have not been widely reported though there are reports on various aspects of alternate bearing such as endogenous and environmental factors [4,18,19]. While the idea of generating a genetically modified olive tree has not been welcome, it is possible to explore molecular mechanisms of the common problems of olive through molecular genetics approaches. Identifying transcription factors specific for important genes of fruit senescence only, for instance, can lead to subsequent steps of controlling these molecules for potentially getting a relatively more uniform harvest. One of the first steps to achieve such long term aims is constructing and characterizing cDNA libraries to identify genes that are specific to certain tissues and / or developmental stages. Furthermore, since the olive genome has not yet been sequenced, olive molecular genetics studies mostly depend on cDNA libraries to identify novel genes, or genes associated with certain processes such as fruit development and senescence.

Although numerous cDNA libraries of various plant tissues and organs under very specific conditions such as phosphorus stressed roots [20], root hairs [21], glucose stressed root tips [22], nodules [23], ripe fruit detachment tissues [24] and leaves [25,26] are available, olive cDNA libraries have largely been restricted to fruit libraries [10,16,17,27,28]. Hence, reports such as comparison of olive genes expressed in leaves of “on year” and that of “off year”, or comparison of olive genes expressed in fruited leaves and non-fruited leaves are rare.

We have recently reported micro RNAs [29] and global transcripts [30] associated with alternate bearing in olive. In this study, olive leaves of trees in “on year” and in “off year” in July (when fruits first appear) and in November (harvest time) were harvested into liquid nitrogen and used to construct cDNA libraries to identify cDNAs specific for each time and condition. Additionally, a fruit cDNA library was also constructed to further confirm the specificity of the cDNAs obtained from each library. Analyses of the results revealed cDNAs specific for each library, and hence, a number of candidate cDNAs associated with alternate bearing were identified. Additionally, bioinformatics tools such as detailed BLAST searches, GenOnthology and KEGG analyzes on the obtained sequences were applied to extract further information about these cDNA molecules.

## Results

### Selection of the reference gene

**Figure 1:**
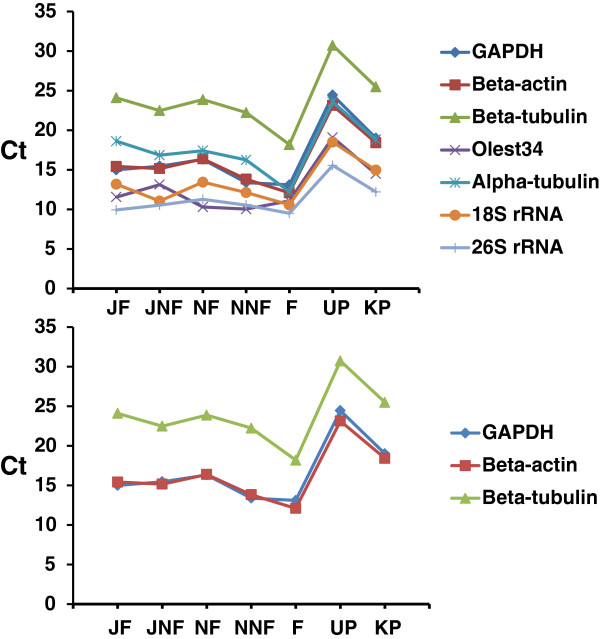
**qRT-PCR amplification of the reference genes evaluated.** Upper panel shows the Ct values of the all 7 reference genes together. The lower panel is simplified from upper panel to display how well GAPDH and beta-actin confirm each other and hence are proper reference genes for olive tissues studied. The tissues used were the same ones used to construct the cDNA libraries except pedicels were collected from Uslu cultivar (UP) and Kiraz cultivar (KP).

Using the appropriate reference gene in quantitative real-time PCR (qRT-PCR) to normalize the initial total RNA template amounts is one of the most important factors affecting the reliability of the qRT-PCR results. That is why commonly used reference genes should be tested first in the organism in use to accurately pick the one that has no variation based on changes because of diverse factors [31]. Since reference genes in olive have not widely been reported for qRT-PCR, expression levels of seven commonly used reference genes (see materials and methods for gene names) in various plants were determined via qRT-PCR (Figure 1) and GAPDH was decided to be an appropriate reference gene to use with olive.

### Brief overview of the cDNAs obtained from the libraries

**Figure 2:**
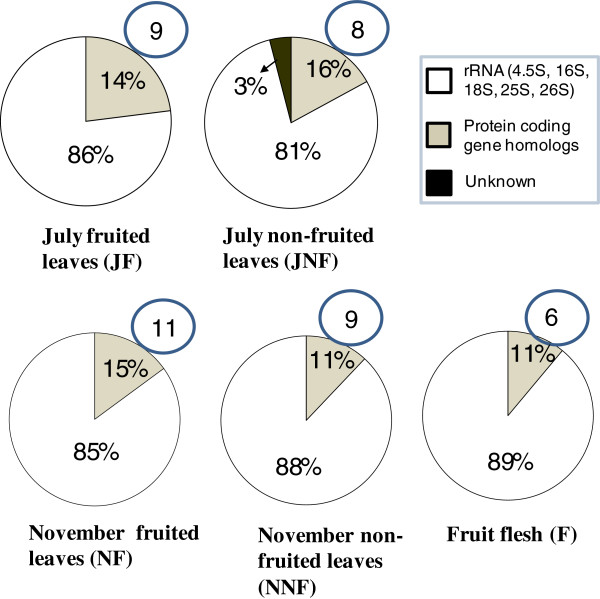
**Overal insert profile of the libraries.** The little circles indicate the number of cDNAs that are new for olive for which 9845 EST records are currently present in GenBank (Databank version: 31.11.2012).

GenBank homologous records for each insert sequence obtained through BLASTn search [32] at NCBI, revealed 11% - 16% protein coding gene homologs and 3% unknown cDNAs while the remaining 84% to 89% constituted non-coding RNA molecules including rRNAs and tRNAs (Figure 2). All the cDNA sequences (except JNF1, JNF32 and JNF87) had similarity to cDNA records of other plants previously registered in the GenBank databases (Tables 1,2,3,4 and 5) while JNF1, JNF32 and JNF87 had no similarity for any records in any available database. In each library, more than half of the cDNAs were new for olive genome (Figure 2).

**Table 1:** cDNA sequences obtained from JF (fruited-leaves in July) and their homologous GenBank records

**cDNA**	GenBankAccn No**	**Size (nt)**	**GenBank record(s) with informing similarity**	**E - Value**	**Primer pairs used in qRT-PCR (All sequences are presented from 5’ to 3’. F: Forward, R: Reverse)**
JF146 JF151 JF187	GW574236	180018201800	*Nicotiana tabacum* cytochrome P450 monooxygenase (CYP72A56) mRNA (gi85068677)	3e-33 8e-34 5e-36	F: TTCTCGTTTGAGATTTCACCTACTTAT
					R: AGAGAGAATGCATAACAACATACGATA
JF124* JF150*	GW574235	1500	*Capsicum annuum* menthone:neomenthol reductase 1 (MNR1) mRNA (gi123691540)	2e-81	F: GGAGTAAGTGTAGAGGGAGATGTCTTA
					R: ACAACCTTAAGAGTGGAATAAATGCTT
JF111* JF148*	GW574234	1000	*Glycine max* transcription factor (bZIP124) mRNA (gi113367217)	3e-52	F: ATCTCCTGGTGCATTTAATTATTGAT
					R: ATCTCCTGGTGCATTTAATTATTGAT
JF154* JF160*	GW574237	3000	*Spinacia oleracea* ClpC protease mRNA, chloroplast gene encoding chloroplast protein (gbAF043539)	0.0	F: TGTGTTAGAACTCTCACTAGAGGAAGC R: CACCATCTAATAACCTGTGTACGAAAT
JF45	GW574239	3000	*O.europaea* putative cytochrome P450 mRNA (gi154257296)	5e-20	F: GAGTACAAGGGACAACATTTTGAGTT R: AGTGGATTCTTCTTCCTCAAAGTTAAT
JF46*	GW574240	550	*Arnebia euchroma* chloroplast protein 12 (CP12) mRNA (gi151564657)	2e-60	F: GTAGGATGTACGTCCACCCAGT
					R: GTTGTCCTTGCAGTAATCTTCCA
JF126*	GW574241	700	*Arabidopsis thaliana* integral membrane HRF1 family protein (At3g59500) (gi145339670)	1e-80	F: TGCAGTCAATTTTATTATTTTGTTTGA
					R: TTTTCCAAATATATTAATGCCAGAACT
JF153*	GW574242	1500	*Arabidopsis thaliana* emryo defective binding / small GTPase regulator (EMB2754) mRNA (gi78498847)	2e-28	F: TTTTATTGTCTGCATTTCTTCAGTTC
					R: ATAAACAGAATTGTCCACCACTACAA
JF178	GW574238	3200	*Raphanus sativus* chloroplast mRNA for ATPase beta subunit (atpB gene) (gi8052351), *Olea europaea* ATP synthase epsilon subunit (embCAD23950)	0.0 1e-31	F: AACTTTCTCAAGATTCTTATTCATCCA
					R: ATAGCACGGAGATCAGTTAATTCAATA

* cDNAs that are new for olive.

** Obtained from GenBank for the sequences generated through this study.

**Table 2:** cDNA sequences obtained from NF (non-fruited-leaves in July) and their homologous GenBank records

**cDNA**	GenBank Accn No**	**Size (nt)**	**GenBank record(s) with informing similarity**	**E - Value**	**Primer pairs used in qRT-PCR (All sequences are presented from 5’ to 3’. F: Forward, R: Reverse)**
JNF1*	GW574243	300	**No signifcant match in any databank**	**-**	F: AACTGACACAATTGCAAAGAGG
					R: CACAGTCATTAATTAACAACCAAAGAA
JNF32* JNF87*	GW574244	1900	**No signifcant match in any databank**	**-**	F: TCACTTCAATATACATGAAACAAAATCTC
					R: AGATAACCAAGAAAAAGAAAGAAGGAG
JNF31 JNF48 JNF84	GW574245	1850 800 1830	*Nicotiana tabacum* cytochrome P450 monooxygenase (CYP72A56) mRNA (gi85068677)	6e-36 8e-33 5e-43	F: TTCTCGTTTGAGATTTCACCTACTTAT
					R: AGAGAGAATGCATAACAACATACGATA
JNF42* JNF82*	GW574246	1000	*Lycopersicon esculentum* ethylene responsive protein (ER6) (gi5669653)	1e-92	F: TGCTCTTCAAATTTGCTCTAATAAAA
					R: TTTGAACAATAGTTGCTAACACTTCC
JNF2 JNF62	GW574247	900	*Lycopersicon esculentum* wound/stress protein (gi51457947)	1e-56	F:AGTGTACCCAGGTAAGTTTCACTGTAG
					R: CAAAGTCCAACAACTCTACATTACAGA
JNF51 JNF55	GW574248	1210 1290	A gene complex of multiple tRNA genes and Photo System II binding proteins (see Accn gi170785617 at NCBI)	0.0	F: CTTGAAATCCAATTCTAAAAGATCAAA
					R: ATAATAGAGGAATGGGGGTAGAGTAGA
JNF56 JNF65	GW574249	1120 1070	*Arabidopsis thaliana* putative ribosomal protein L10 (At1g08360) (gi21386914)	0.0	F: CAAAAACTATGATCCACAGAAGGATA
					R: CTTAACCAGTTTCTTGTTTTTGTTCA
JNF4	GW574250	1000	*Zea mays* disease resistance response protein (gi100284658)	0.0	F: GGACATGTTGTTTTGGAATATACTCTT
					R: CATAACAACCTAAAGATGAAAAGAACC
JNF79*	GW574251	3000	*C.roseus* cpr mRNA for NADPH ferrihemoprotein reductase (gi18138)	0.0	F: TCAAACTTCCTGCTGATTCTAAAGT
					R: AAAGTTGTTCAACTCATCCTCGTAG
JNF83*	GW574252	900	*Populus trichocarpa*, unknown mRNA (gi18485578)	3e-25	F: GCGTAGTGACTAATCCTTGTCTACC
					R: AAGTTGGTAAAAGACAAATTTCAACAT
JNF92	GW574253	1600	*Solanum phureja* DnaJ-like protein isoform (gi113374277)	1e-43	F: TGATTGAGATCAAACAAGCATACAG
					R: ATGTCTAAATCGTACAACGCTCTCTT
JNF96*	GW574254	1000	*Arabidopsis thaliana* ATHX (THIOREDOXIN X) mRNA (gi45336578)	1e-36	F: TTGAAAAATACAAGGTATATGGATTGC
					R: TCTAAGTAACAGACACAGACCTCAGAA

* cDNAs that are new for olive.

** Obtained from GenBank for the sequences generated through this study.

**Table 3:** cDNA sequences obtained from NF (fruited-leaves in November) and their homologous GenBank records

**cDNA**	GenBank Accn No**	**Size (nt)**	**GenBank record(s) with informing similarity**	**E - Value**	**Primer pairs used in qRT-PCR (All sequences are presented from 5’ to 3’. F: Forward, R: Reverse)**
NF3 NF96	GW574256	900	*Nicotiana sylvestris* cryptochrome 1 (cry1) mRNA (gi78217440)		F: AAACGGTTAGAACCATCAATACTTTC
					R: CCAGTGAATTGATCAGAGAAATTAGA
NF17* NF90*	GW574257	1780	*Arabidopsis thaliana* DNAJ heat shock family protein (At2g22360) mRNA (gi42569238)	1e-67	F: ATGCTGAAGAGAAGTTTAAGGAGATTAG
					R: TCAAATAATGACTCAAATAGATCAAAGG
NF69* NF99*	GW574258	700	*Arabidopsis thaliana* PSI-N; calmodulin binding (PSAN) mRNA (gi145359627)	2e-67	F: GTACCATATATTTCTGAGGACTTGGAG
					R: GTTTAGAAGTTGCAAGTGGAAAAATAG
NF2*	GW574259	900	*Avicennia marina* dehydrin (DHN) mRNA (gi157497150)	2e-28	F: ATGAAGGAACTTACGACACTTCAAC
					R: TAATACAAACATGAAAAAGCACACG
NF8*	GW574260	1000	*Lycopersicon esculentum* transcription factor JERF1 (JERF1) mRNA (gi22074045)	5e-55	F: GAGAAACCGCCAACAAATAAGTATAG
					R: ATTTCTGGAGTTTTAGCACAATTTTC
NF9	GW574261	400	*Avicennia marina* class I type 2 metallothionein mRNA (gi12963447)	2e-41	F: GAATTGTATGAATGTTTTGGGTAAATC
					R: TTGGTTTTTCGGTATATAATTAAGCAG
NF22	GW574262	1000	A gene complex of multiple tRNA genes and Photo System II binding proteins (see Accn gi170785601 at NCBI)	0.0	F: GCCTCTAGGAATTTCTGGTACTTTC
					R: GTAACCTGCATTAGCAGATTCATTT
NF37*	GW574255	1180	*Arabidopsis thaliana* EMBRYO DEFECTIVE 2734 lyase mRNA (gi30687496)	2e-85	F: CTGGTTGAGCTGCTTACCTATAAAA
					R: TTGCTCTGTAGCAAGATCTTTACCT
NF41*	GW574263	2000	*Arabidopsis thaliana* splicing factor, putative (At5g64270) mRNA (gi30697984)	0.0	F: TTATTGAACATGGTCTTAATGATGAAA
					R: ATATATGGCATCCATAAGTGGTATGAT
NF58*	GW574264	4000	*Arabidopsis thaliana* glycosyl hydrolase family 38 protein (At5g13980) mRNA (gi79598780)	1e-99	F: TTTATTAAGAAGGAGTTTGGTGTGACT
					R: AACGACTTCAAGACTCTCTCATATTTC
NF60*	GW574265	2000	*Arabidopsis thaliana* AtEXO70E2 binding protein mRNA (gi30697462)	7e-09	F: ATGAGGTAGTGAAAGAAGATGGACTTA
					R: ATTATTTAGGCTCAATCTCTCCAAACT
NF95*	GW574266	3000	*Datura stramonium* mRNA for arginine decarboxylase 1 (adc1 gene) (gi6646839)	2e-54	F: CTAATCACCCTTCCAAGATTCTTTACT
					R: AGAAAGCGTGGAGTATGAGTAGTATGT

* cDNAs that are new for olive.

** Obtained from GenBank for the sequences generated through this study.

**Table 4:** cDNA sequences obtained from NN (non-fruited leaves in November) and their homologous GenBank records

**cDNA**	GenBan Accn No**	**Size (nt)**	**GenBank record(s) with informing similarity**	**E - Value**	**Primer pairs used in qRT-PCR (All sequences are presented from 5’ to 3’. F: Forward, R: Reverse)**
NNF14* NNF36* NNF85*	GW574267	1430 1360 1410	*Arabidopsis thaliana* zinc finger family protein (At2g31770), IBR domain -containing protein (gi91806300)	0.0	F: CAGGAATCAAAAGAAAATATTCTGAAC
					R: TCGACTCATAGACAAACTATGTACAGG
NNF22*	GW574269	1100	*Vitis vinifera* hypothetical protein mRNA (gi225450142)	2e-22	F: CAAATCTCTTCATCTTCTTCAATTCTG
					R: CAGTATAAAAACTTGATTCCCTCCATA
NNF23*	GW574270	1000	*Lycopersicon esculentum* temperature - induced lipocalin mRNA (gi77744858)	0.0	F: ATATAAGTCTGATCCCAATAGTGACGA
					R: TTCTGTACAAGCATGTAGTGTATCTCC
NNF24*	GW574271	1200	*Nicotiana tabacum* eIF4E (initiaition factor) mRNA (gi51599168)	1e-63	F: GAATGTCAGATTAAGGCAGGATAAA
					R: AGCTTCTTAGCATCCTCATGAAATA
NNF29*	GW574272	1670	*Arabidopsis thaliana* transducin family protein (At2g26490) mRNA (gi42569344)	1e-74	F: GGTTGTGTATAGTGGGAGTCTTGATA
					R: ACTTTGATTTGATCAATACTTTGCTG
NNF31	GW574273	800	*Plantago major* cold stress-induced protein (src1 gene) mRNA (gi53748474)	7e-27	F: AAAAAGAAGAAAGACAAGAAAAAGCAT
					R: ATACACAATCAAAGAGTAGCCAACAAC
			*Retama raetam* drought-induced protein (DIP) mRNA (gi16198345)	1e-24	
NNF32	GW574274	600	Multiple tRNA genes and PSII binding proteins (see Accn gi170785617)	0.0	F: CTTGAAATCCAATTCTAAAAGATCAAA
					R: ATAATAGAGGAATGGGGGTAGAGTAGA
NNF59*	GW574275	800	*Arabidopsis thaliana* PSRP4 (Plastid specific-ribosomal protein 4) mRNA (gi145360741)	3e-39	F: ATTCTCTCACATCAATCTCATCTCC
					R: GTCCTCTTCCCTTATTCTTGTCCT
NNF91*	GW574268	660	*Arabidopsis thaliana* pre-mRNA splicing factor (At4g14342) mRNA (gi145361320)	4e-56	F: TTCTATCGGGTGTATAATTTGATCTTT
					R: TTATTAACCAAGTGGGTACAGATTCTT

* cDNAs that are new for olive.

** Obtained from GenBank for the sequences generated through this study.

**Table 5:** cDNA sequences obtained from F (fruits in November) and their homologous GenBank records

**cDNA**	GenBank Accn No**	**Size (nt)**	**GenBank record(s) with informing similarity**	**E - Value**	**Primer pairs used in qRT-PCR (All sequences are presented from 5’ to 3’. F: Forward, R: Reverse)**
F46* F55* F57*	GW574276	132014201450	At2g31770 zinc finger family protein (gi91806300), *Arabidopsis thaliana* IBR domain-containing protein / ARIADNE - like protein ARI7 mRNA	2e-60 0.0 2e-76 0.0 2e-66 0.0	F: CAGGAATCAAAAGAAAATATTCTGAAC
					R: TCGACTCATAGACAAACTATGTACAGG
F4 F22	GW574277	12501240	*Petunia* x *hybrida* mRNA for triosephosphate isomerase	3e-81 0.0	F: CCTGGATTAACTTGTGCATTTATACTT
					R: CATCTAAGCGAAGTTCCAAATAGATAC
F12* F20*	GW574278	1440	*Solanum tuberosum* UDP-glucose 4-epimerase (StUGE45) mRNA	0.0	F: TATATTGCTGAGGTACTTCAATCCAG R: TGCTAAATCCACAACATGGATATAAT
F13 F17	GW574279	510	*Digitalis lanata* mRNA for Acyl-CoA binding protein (acbp4 gene)	4e-74	F: CAAGCTTGTTCTTTATGGACTTTACA R: CATGGATGAGTACTTAGTTATGCTGCT
F10*	GW574280	3000	*Arabidopsis thaliana* nuclear-pore anchor	3e-14	F: GTCGTCTTCCCAAAATATAGAAACTC R: GGGTCTCTACACCTTTAGACTTTTTG
F51	GW574281	800	Multiple tRNA genes and PSII binding proteins (see Accn gi170785617)	0.0	F: CTTGAAATCCAATTCTAAAAGATCAAA R: ATAATAGAGGAATGGGGGTAGAGTAGA

* cDNAs that are new for olive.

** Obtained from GenBank for the sequences generated through this study.

### cDNA contents and qRT-PCR validation of the individual libraries

**Figure 3:**
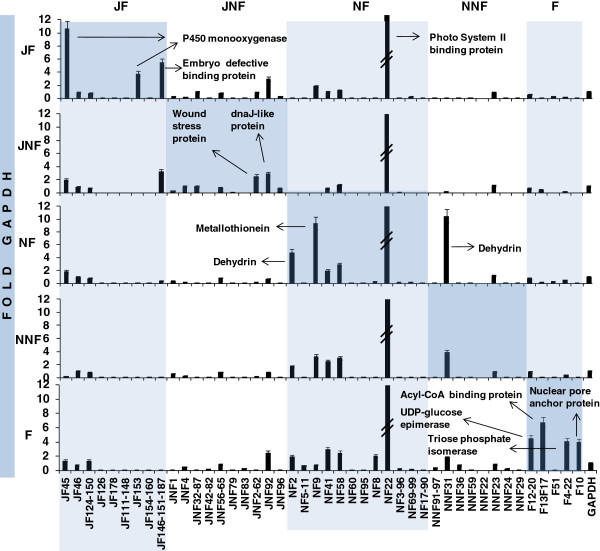
**qRT-PCR analysis of the cDNAs obtained from all libraries in this study.** The cDNAs were separately amplified from each tissue of which cDNA libraries were constructed (Five separate plots are horizontally aligned). Dark shaded boxes highlight the cDNAs that had the highest expression in tissues where they were detected through cDNA library screening. Light shaded columns and unshaded columns highlight cDNAs obtained from a specific library. Highly expressed cDNAs from each library were labeled. Expression levels are average values of at least 3 reactions. Error bars are indicated for each cDNA. JF: July fruited leaves, JNF: July non-fruited leaves, NF: November fruited leaves, NNF: November non-fruited leaves, F: Fruits. NF22 expressed at least 37 fold GAPDH in each library and therefore it was marked with an interruption sign. NNF22 was not included in the qRT-PCR analyses. The bars lower than 1 correspond to expression level that is less than that of GAPDH. The absence of bars is due to too low expression levels to show on the graph.

In July, “on year” leaves (JF), 4% of the protein coding gene homologs was detected as P450 monooxygenases (three and one, gi85068677 and gi15425796, respectively) while homologs of menthone:neomenthol reductase 1, a transcription factor (gi113367217) and a protease (gbAF043539) were represented by 2% (Table 1). The remaining five cDNAs were represented with 1% (Table 1). qRT-PCR confirmed the abundance of P450 monooxygenases mentioned in JF library, and revealed embryo defective binding / small GTPase regulator (gi78498847) to be specific to “on year” leaves in July (Figure 3).

In July “off year” leaves (JNF), the predominating cDNAs were (JNF31, JNF48, JNF84) again homologs of P450 monooxygenase (gi85068677) that was also confirmed by qRT-PCR (Figure 3). The second most abundant cDNAs were homologs of ethylene responsive protein (gi5669653), wound stress protein (gi51457947), a gene complex of multiple constitutive proteins and intergenic spacers (gi17085617, similar to gi170785601 and also detected once in each of NNF and F) and ribosomal protein L10 (gi21386914). Only in this library (JNF) were detected cDNAs (JNF1, JNF32, and JNF87) that were completely novel to any available nucleotide database. qRT-PCR revealed wound stress protein (gi51457947), one of the abundant cDNAs in JNF library, as a specific cDNA to “off year” leaves in July.

Although the abundance of the cDNAs detected in November, “on year” leaves (NF) were the least (no more than 2%) among all libraries generated (Table 3), NF2 (a putative dehydrin), NF9 (a putative metallothionein) and a cDNA from JNF library (JNF31, a putative cold stress induced protein / dehydrin) appeared to express at very high levels (5, 9 and 11 fold GAPDH respectively). Homologs of cryptochrome 1 (cry1) mRNA, DnaJ heat shock family protein (gi42569238), and a calmodulin binding protein (gi145359627) were detected as 2% (Table 3) but their abundance was not confirmed by qRT-PCR.

A cDNA that is homologous to both ARIADNE (gi145360514, a ubiquitin - protein ligase) and a zinc finger family protein (gi91806300) was the most abundant cDNA of the remaining two libraries (NNF and F). It was detected at a rate of 3% in each library (Table 4, Table 5). qRT-PCR analysis revealed this cDNA’s expression as 6 fold to 11 fold more in F library than in any other library (Figure 3). The remaining cDNAs in the NNF library were detected only once (Table 4). The cold stress-induced protein / dehydrin homolog (NNF31) along with the other putative dehydrin (NF2) and the putative metallothionein (NF9) were the most abundant cDNAs in both of the November libraries (NF and NNF) from the leaves (Figure 3).

After ARIADNE - like protein homologs, the second most abundant (2% each) cDNAs in fruit flesh library (Table 5) were homologs of UDP-glucose 4-epimerase (gi37781555), acyl-CoA binding protein (gi6002103) and triosephosphate isomerase (gi602589). qRT-PCR revealed all the cDNAs (except F51 which is similar to a PSII binding protein) isolated from fruit flesh library were specific to fruits. A cDNA (F10) that has a weak similarity to predicted nuclear-pore anchor protein (gi844268) was also fruit specific (Figure 3).

### Bioinformatics

**Figure 4:**
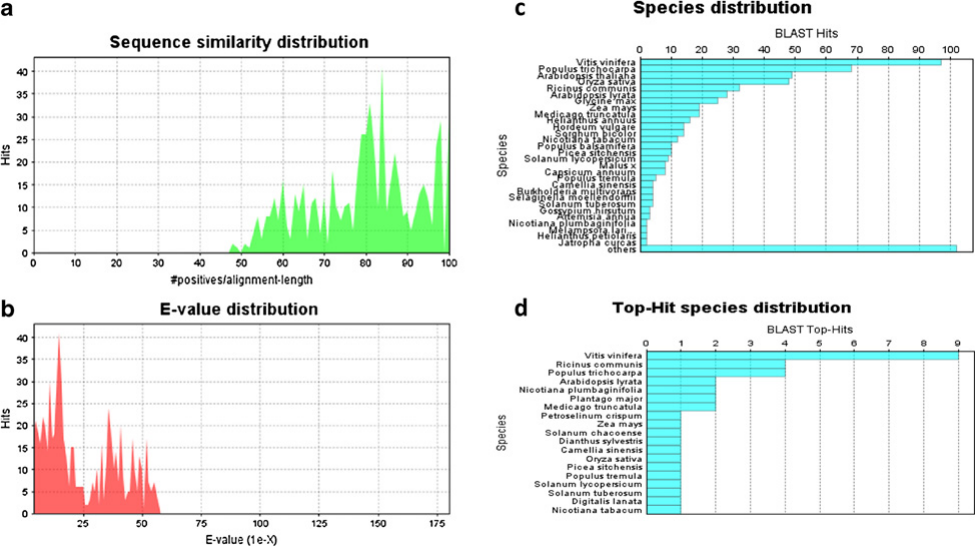
Sequence similarity (a), E-value distribution (b), species distribution (c) and top-hit species distribution (d) graphs generated after olive sequences were processed.

**Figure 5:**
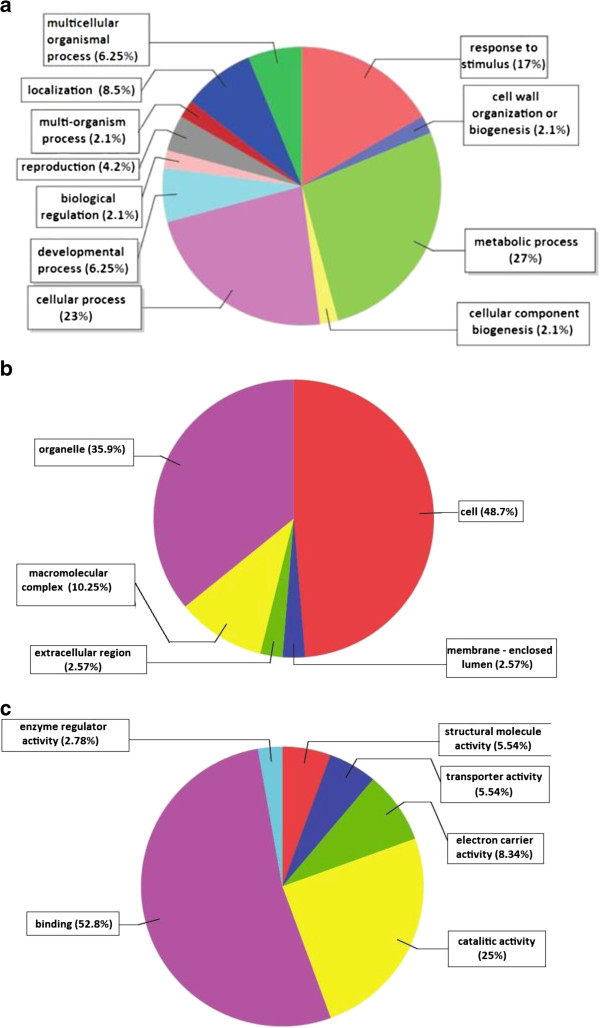
**The pie diagrams demonstrating the percentage share of putative olive transcripts within the functional categories of Gene Ontology (Biological Process, Cellular Component and Molecular Function) based on the Blast2Go data mining.** (**a**) The GO terms of olive transcripts for Biological Process; 1-Metabolic process, 2-Cellular process, 3- Response to stimulus, 4-Localization, 5-Multicellular organismal process, 6-Developmental process, 7-Reproduction, 8-Multi-organism process, 9-Biological regulation, 10-Cell wall organization, 11-Cellular component biogenesis. (**b**) The GO terms of olive transcripts for Cellular Component; 1-Cellular, 2-Organelle, 3-Macromolecular complex, 4-Extracellular region, 5-Membrane enclosed lumen. (**c**) The GO terms of olive transcripts for Molecular Function; 1- Binding, 2-Catalytic activity, 3-Electron carrier activity, 4-Transporter, 5-Structural molecule activity 6-enzyme regulator activity.

Among the olive ESTs analyzed, 35 sequences displayed significant BLASTx matches within the genes registered in the NCBI database. In order to predict the reliability assessment of the ESTs or alignment quality, sequence similarity (Figure 4a) and E-value distribution graphs (Figure 4b) were generated from sequences based on the BLASTx results. The species distribution of olive ESTs based on BLASTx hits had the highest sequence homology to *Vitis vinifera* (~96%), and followed by *Populus trichocarpa* (~68%), *Arabidopsis thaliana* (~48%) and *Oryza sativa* (~47%) (Figures 4c, 4d) (see Additional file 1). The functional annotation and categorization of each olive EST based on Gene Ontology (GO) terms were analyzed using the Blast2GO suite. For each transcript, a set of GO term information including; accession, annotation term and basic definition is shown in table (see Additional file 2). In addition, these transcripts representing genes with known function were categorized by biological process, cellular component and molecular function according to the ontological definitions of the GO terms. The transcripts in the biological process (Figure 5a) category were related to metabolic process (27%), cellular process (23%), response to stimulus (17%), cellular localization (8.5%), multicellular organismal process (6.25%), developmental process (6.25%), reproduction (4.2 %), multi - organism process (2.1%), biological regulation (2.1%), cell wall organization or biogenesis (2.1%) and cellular component biogenesis (2.1%). In the cellular component category (Figure 5b), most of the GO terms were mainly related to cellular (48.7%) and organelle (35.9%) components such as cell periphery and intracellular organelle parts, followed by macromolecular complex (10.25%), extracellular region (2.57%) and membrane enclosed lumen (2.57%). As for the molecular function (Figure 5c) category, most abundant GO terms were involved in binding (52.8%) such as nucleic acid and transition metal ion binding, catalytic activity (25%), electron carrier activity (8.34%), transporter (5.54%) and structural molecule (5.54%) activity as well as enzyme regulator (2.78%) activity (For all functional categories, the pie charts and sequence distribution tables pertaining to the olive ESTs are presented in the Additional file 3). Using KAAS, each olive EST was assigned with a KEGG orthology (KO) number with the SBH (single-directional best hit) assignment method and the numbers subsequently were mapped to one of the KEGG's reference metabolic pathways. Consequently, a total of 13 main metabolic pathways were generated through the use of KAAS pathway mapping and the sequences largely correlated with essential metabolisms of galactose metabolism (1) , amino sugar and nucleotide sugar metabolism (1), photosynthesis (1), other glycan degradation (1), monoterpenoid biosynthesis (1) and followed by spliceosome (2), ribosome (2) and circadian rhythm - plant (1) (Table 6, and detailed information and images about the pathway found in the Additional file 4.

**Table 6:** Ortology number, metabolic pathway and maps entries of olive ESTs involved in the main metabolic processes

Sequence Code*	**Kegg Orthology Number**	**KEGG maps entry**	**Metabolic Pathway**
F12F20	ko00052	K01784	Galactose metabolism galE; UDP-glucose 4-epimerase
F12F20	ko00520	K01784	Amino sugar and nucleotide sugar metabolism galE; UDP-glucose 4-epimerase
NF22	ko00195	K02703	Photosynthesis psbA; Photosystem II P680 reaction center D1 protein
NF58	ko00511	K01191	Other glycan degradation Alpha-mannosidase
JF124JF150	ko00902	K15095	Monoterpenoid biosynthesis (+)-neomenthol dehydrogenase
NF41 NNF91NNF97	ko03040	K12828 K12832	Spliceosome SF3B1; splicing factor 3B subunit 1 Spliceosome SF3B5; splicing factor 3B subunit 5
JNF56JNF65	ko03010	K02865 K02913	RP-L10Ae; large subunit ribosomal protein RP-L33; large subunit ribosomal protein L33
NF3NF96	ko04712	K12118	Circadian rhythm – plant CRY1; cryptochrome 1

*See Tables 1–5 for the accession numbers of these sequences.

According the table, there is a correlation between the biosynthesis (galactose, amino sugar and nucleotide sugar, monoterpenoid and photosynthesis) and ribosomal activity in fruited and non- fruited leaves

## Discussion

### The approach to isolate differentially expressed genes

To isolate differentially expressed cDNAs, it is essential to start with total RNA molecules extracted from tissues of identical conditions. With this respect, the trees we selected were genetically identical and grew virtually in the same micro-environment. The same constitutive cDNA (gi170785601 / gi170785617, a gene complex of multiple tRNA genes and Photo System II binding proteins) was detected in four (all except JF) of the five libraries (Tables 1,2,3,4 and 5) and was confirmed with qRT-PCR while homologs of cDNAs reported to express at certain stress conditions such as cold stress induced protein / dehydrin (NNF31) and temperature induced lipocalin (NNF23) or at certain metabolic processes such as Acyl-CoA binding protein (F13F17) and triosephosphate isomerase (F4F22) were detected at the expected libraries (Table 4, Table 5). At least 2 cDNAs from each library (except NNF) were confirmed by qRT-PCR to be specific to the tissues (leaves of JF, JNF, NF, NNF and fruits of F) that they were initially detected through sequencing of the plasmid inserts from arbitrarily selected colonies. Hence, the approach (of using total RNA to prepare cDNA libraries, instead of purified mRNA pool) we used to detect differentially expressed cDNAs in the libraries, has proven to be reasonable. Furthermore, more than half of the cDNAs of each library did not match olive records in nucleotide (2718 sequences) and EST (9845 sequences) databases of NCBI which contains cDNAs derived from leaves, fruits and flowers (Database version: 31.11.2012).

### Overall cDNA profile of the libraries

In July, “on year” leaves (JF) and in “off year” leaves (JNF), homologs of cytochrome P450 monooxygenases (JF45 and JF146/JF151/JF187) appeared to be dominating cDNAs (Table 1, Table 2, Figure 3). Embryo defective binding / small GTPase protein homolog (JF153) appeared to be strictly specific to JF. Interestingly, JF45 was detected more in “on year” leaves and fruits but less in “off year” leaves (Figure 3) suggesting it might have a role in “on year” but more in July than that of November. On the contrary, the putative metallothionein (JF9) appeared to be associated with “on year” leaves but more in November. One (JNF1) of the two unknown cDNAs isolated from July “off year” leaves were found to express in all the tissues studied except fruits while the other (JNF32JNF87) was specific to July leaves only.

In November, “on year” leaves (NF), a homolog of EMB lyase (gi30687496) was the most abundant cDNA (4%), yet it was detected in this library only. Based on the NCBI record (EMB2734), this putative lyase is predicted to function in breaking of C-C, C-O and C-N bonds during embryo development. Detecting this cDNA in maturing fruit bearing leaves makes meaningful sense as for developing embryo (as a sink tissue), nutrients from the leaves (as source tissues) should be supplied [33]. Most other cDNAs of November “on year” leaves (NF) also appeared to be associated with cold stress and embryo development which were the specific conditions for NF library: NF2 is a homolog of dehydrin (gi157497150) that has been reported to function in low temperatures and seed development [34]. NF9 is similar to metallothionein (gi12963447) and has been reported to function in senescence [35,36]. Likewise NF8 homolog JERF1 (gi22074045) has been reported to involve in gene expression at cold [37], and NF58 homolog glycosyl hydrolase (gi79598780) is associated with biotic / abiotic stress, lignification and cell wall reconstruction [38].

The cDNA that is homolog of both ARIADNE (gi145360514, a ubiquitin-protein ligase) and a zinc finger family protein (gi91806300) was the most abundant in both November, “off year” leaves (NNF) and in fruit flesh (F) libraries. qRT-PCR results revealed 6 fold to 11 fold more expression of this cDNA in fruits than in other libraries but did not confirm as one of the most abundant cDNAs in fruits nor in “off year” leaves. Combined with ubiquitin association, these results suggest ARIADNE homolog in olive is most probably a constitutively expressed cDNA. NNF91 / NNF97 and NNF24 are homologs of a splicing factor subunit (gi91806300) and a translation initiation factor (gi51599168), respectively, and they both were detected at very low level (less than 0.3 fold GAPDH) in all libraries (Figure 3).

Given the fact that these two trees are genetically identical and grow virtually in the same micro-environment, overall results present cDNAs differentially expressed in leaves, “on year” leaves, and in “off year” leaves. Constitutively expressed genes, most of which have not been detected in olive before, and several unknown cDNAs and / or genes are also reported. It should be kept in mind that alternate bearing is a result of complicated biotic and abiotic processes including environmental factors, physiological responses of the trees in the form of activation and repression of endogenous metabolic pathways [18,19,39-41], which in turn are also based on the genetic background of the tree. Large phenotypic variation has been observed, including year-to-year variation of a single genotype, as well as variation among and within (multiclonal) cultivars under the same environment. Hence it is not possible to clearly enlighten the genetic players of alternate bearing in a single cDNA screening or even a complete transcriptome analysis. Multiple approaches involving several years follow up of the selected trees / cultivars are needed to identify certain or key genetic players of alternate bearing in olive. There are no comprehensive reports on the genetic basis of alternate bearing in olive, however, and hence these results constitute important information for one of the first steps of a genetic dissection of olive periodicity which causes significant economic loss for olive growers. Through exploring these cDNAs further, it is possible to isolate genes that are key regulator of fruit formation and / or periodicity in olive.

### Bioinformatic analyses

Through the bioinformatic analyses it was possible to extract further additional information about the cDNAs as well as about olive in general. BLASTx analysis revealed olive has a surprisingly high (96%) similarity to grapevine (*Vitis vinifera*), although these two plants are not even the same order (*Vitis* in Vitales while *Olea* in Scrophulariales) in systematics. The second most similar plant to olive is *Populus* (a tree with no fleshy fruits) with a much lower (68%) similarity. This suggests the cDNAs captured are directly or indirectly associated with the pathways of fruit formation and / or production that are in turn related to periodicity. GO terms categorization grouped the cDNAs into common processes, localizations and functions such as metabolic process, cellular localization and binding, respectively, which reflect a general profile of typical cell while differentially expressed cDNAs were also significantly represented such as 17% of the cDNAs in the “response to stimulus” category, and 25% of the cDNAs in the “nucleic acid and transition metal ion binding, catalytic activity” category. The metabolic pathways generated through the use of KAAS pathway mapping were largely correlated with essential metabolisms such as galactose, amino sugar, nucleotide sugar metabolisms and photosynthesis confirming the constitutive status of the majority of the cDNAs obtained.

## Conclusions

In summary, we have isolated and analyzed cDNAs that are associated with alternate bearing in olive. A P450 monooxigenase homolog expressed more in the “on year” than that of “off year” leaves in July. Two putative dehydrins expressed significantly more in “on year” leaves than that of “off year” leaves in November. Homologs of triose phosphate isomerise, UDP - glucose epimerase, acyl - CoA binding protein, and a putative nuclear core anchor protein appeared fruit specific, while a homolog of an embryo binding protein / small GTPase regulator was detected in “on year” leaves only. An unknown cDNA was specific to leaves in July. KEGG pathway analyses of the sequences correlated with essential metabolisms such as galactose metabolism, amino sugar and nucleotide sugar metabolisms and photosynthesis. Detailed analysis of the results presents candidate cDNAs that can be used to dissect further the genetic basis of fruit production and / or alternate bearing.

## Methods

### Experimental design and the confirmation of the genetic identity of the individual trees

Two side by side olive (*Olea europaea* L. cv. Ayvalık) trees (approximately 4 m apart from each other), one in “on year” (high fruit yield) and one in “off year” (almost no fruits on the tree), were picked in Gömeç Orchard of Edremit Olive Seedling Growing Station. The trees (about 5 m high) were transferred into soil around 15 years ago from scions that were taken from the same tree. The scions had first dipped into indole butyric acid and then rooted in sandy soil before they were transferred into soil. Leaves from “on year” tree and from “off year” tree were randomly collected and separately deposited (for each tree) in liquid nitrogen and directly (or after keeping in - 80°C freezer until use) used for total RNA extraction. Total RNA extraction from fruits and pedicels were conducted as described above. To make sure the selected two trees have the same genetic identity, total genomic DNA (gDNA) was isolated using Plant DNeasy Kit (Qiagen, Germany) and used as template for PCR reactions to amplify JNF96, NF2 and NNF31 separately from these two trees. PCR products were then sequenced at RefGen (Gen Araştırmaları ve Biyoteknoloji, Ankara) using an ABI 3130XL Genetic Analyzer (Applied Biosystems, Fostercity, CA) with a BigDye Cycle Sequencing kit (Applied Biosystems, Fostercity, CA). JNF96, NF2 and NNF31 were proved to have unique DNA sequence in 29 olive cultivars tested (unpublished data) and hence were utilized as markers to determine the genetic identity of the two trees used in this study. Comparison of the sequences revealed no nucleotide differences (100% identical) for any of the three markers between the two trees, and hence their genetic identity was confirmed.

### Construction of cDNA libraries

Total RNA extraction was performed using RNeasy Kit (Qiagen, Germany) following manufacturer instructions. RevertAid H minus 1st Strand cDNA Synthesis Kit (Fermentas, Lithuania) was used to synthesize the first strand cDNA molecules which were then incubated with RNase H (Fermentas, Lithuania) to remove RNA strand of DNA - RNA hybrids. The second strands were synthesized with DNA Polimerase I (Fermentas, Lithuania). Fifteen units of T4 DNA Polimerase (Fermentas, Lithuania) was used for blunting the double strand cDNA molecules which were then column - purified with a PCR Purification Kit (Qiagen, Germany) and cloned into pJET1.2 (Fermentas, Lithuania) using CloneJET™ PCR Cloning Kit (Fermentas, Lithuania). Manufacturers’ protocols of the kits were followed in each reaction. Glycerol stocks were prepared for each colony that was confirmed to harbor an insert bearing plasmid (pJET1.2) through restriction digestion (of 100 randomly picked colonies from each library) with *Bgl*II (Fermentas, Lithuania). Plasmids from insert - positive clones were isolated using GeneJET^TM^ Plasmid Miniprep Kit (Fermentas, Lithuania) and sequenced at RefGen (Gen Araştırmaları ve Biyoteknoloji, Ankara) using an ABI 3130XL Genetic Analyzer (Applied Biosystems, Fostercity, CA) with a BigDye Cycle Sequencing kit (Applied Biosystems, Fostercity, CA). Since detecting the most abundant genes of each specific condition (such as “on year” leaves or “off year” leaves) was the aim of the study, it was reasoned that the non-coding RNAs (rRNAs and tRNAs) should not be removed when preparing the first strand cDNA templates. Therefore total RNA (instead of isolated mRNA) was intentionally preferred for library construction to detect significantly abundant genes in each specific condition. Oligo dT primers were used instead of random oligos, however, to increase the number of protein coding cDNAs detected. Obtaining around 15% protein coding gene homologs versus around 85% non coding RNA (rRNA and tRNA) from each library on average (Figure 2) suggested that the approach was reasonable. The five cDNA libraries constructed were named as JF (July, “on year” leaves), JNF (July, “off year” leaves), NF (November, “on year” leaves), NNF (November, “off year” leaves) and F (Fruit flesh).

### Quantitative real-time PCR analysis of cDNAs

To confirm the spatial and temporal expression status of cDNAs, qRT-PCR was conducted on a Rotor-Gene 6000® (Qiagen AG Hilden, Germany) using FastStart Universal SYBR Green Master (Roche Mannheim, Germany) for all the cDNAs obtained. qRT-PCR reaction for each gene was run at least in triplicates and repeated when a deviation more than 1 Ct (cycle threshold) was observed. Hence the Ct values were obtained by averaging at least of three different reactions. Cycling conditions were set as one cycle of 95°C for 5 minutes followed by 35 cycles of 94°C for 20 seconds, 50°C for 20 seconds and 72°C for 20 seconds. After trying Olest34 (dbEST Id: 20623527), alpha-tubulin (GenBank ID: 154259491), beta-actin (GenBank ID: 23477718), 26S rRNA (GenBank ID: 19919612), 18S rRNA (GenBank ID: 17028035) and Glyceraldehyde phosphate dehydrogenase (GAPDH, GenBank ID:154260889) of olive in qRT-PCR, GAPDH was found to be the least spatially and temporally variable gene, and hence was used to normalize the copy numbers of the cDNAs tested. The primer pairs used for these cDNAs are as follows: GAPDH-F: 5'-ACA GCT CCT GGT AAG GGT GA-3', GAPDH-R: 5’-GGC TTG CGT CAA GAA GTC TC-3', Olest34-F: 5'-GAC CGT AGG TGC GAT GAT TT-3', Olest34-R: 5'-CCG CCT GGA CAA TTA GAC AT-3', alpha-tubulin-F: 5'-TCA CTG GTA TGT GGG TGA GG-3', alpha-tubulin-R: 5'-TGA GAC CAT TGG CTT GAT TG-3', beta-actin-F: 5'-GAA TTG CCA GAT GGA CAG GT-3', beta-actin-R: 5'-GAA CCA CCA CTG AGG ACG AT-3', beta-tubulin-F: 5'-CCG GTA CAA AGC GAC AAT GAT-3', beta-tubulin-R: 5'-AGG GGA TGG GAA GAC AGA GAA AGT-3', 26SrRNA-F: GAC TTA GAG GCG TTC AGT CAT AAT C-3', 26SrRNA-R: 5’-GTG AGA CAG GTT AGT TTT ACC CTA CTG-3’, 18SrRNA-F: ATT TAA GTT GTT GCA GTT AAA AAG CTC-3', 18SrRNA-R: GCA CTC TAA TTT CTT CAA AGT AAC AGC-3'. The primer pairs used to amplify the cDNAs obtained from all libraries are shown in their respective tables (Tables 1–5).

### Bioinformatics analyses of cDNA sequences

Insert (cDNA) sequences obtained from RefGen (Ankara, Turkey) were analysed using FinchTV v1.4 (Geospiza, Seattle, WA) and BioEdit [42] for chromatogram quality and contig construction. Insert sequences confirmed for accuracy were blasted [32] in BLASTn and BLASTx databases of NCBI - GenBank, and the homologous records from other plants were determined. When no significant hits from BLASTn and BLASTx databases were obtained, all other available databases were searched and the significant hits were recorded. Putative identities and functional annotation of the unique sequences obtained from the leaves (“on” and “off” years) and fruits were determined using the Blast2Go (B2G) software suite v2.3.1 with the default parameters (http://www.blast2go.com/b2ghome) [43]. The annotation process of assembled cDNA sequences (contigs) was mainly performed in three steps; (i) sequence similarity search of each individual ESTs was compared to the sequences in non-redundant database in GenBank by using the BLASTx algorithm with default settings, (ii) gene mapping and gene ontology (GO) categorization of unique sequences based on the BLASTx hits, and (iii) KEGG (Kyoto Encyclopedia of Genes and Genomes) was used to decipher the biological function of unique sequences. After processing EST sequences such as sequence cleaning, vector masking and clustering, all ESTs were converted into fasta format. First, a set of olive EST sequences in fasta format were loaded into the B2G software and homology analysis against the NCBI nr protein database was conducted using a BLASTx algorithm (cutoff E-value of 1.0E-5 ) in order to identify the gene and protein names pertaining to the olive ESTs. Sequences having no homology (or BLASTx hits) to the protein database were further analyzed at the nucleotide level with BLASTx (cutoff E-value of 1.0E-5). The detailed BLAST results were automatically extracted and converted into excel tables including; sequence length, gene name, e-value, similarity, hit-length, align-length, GenBank and Uniprot accession numbers as well as Gene Ontology IDs belonging to each sequences (see Additional file 5). During the annotation process, each olive EST was analyzed to address the functionality of newly identified genes using the KEGG automatic annotation server (KAAS-http://www.genome.ad.jp/tools/kaas/) which provides the functional annotation of genes based on sequence similarity comparisons against the genes within KEGG Genes database [44].

## Competing interests

The authors declared no conflict of interest concerning the work in this paper.

## Authors’ contributions

ED designed the research, conducted and analyzed the experiments, wrote the manuscript. OS planned and performed experiments. TU planned and performed the bioinformatics analyses and critically evaluated the manuscript. AD helped collecting and processing the materials and critically evaluated the manuscript. All authors read and approved the final manuscript.

## Supplementary Material

Additional file 1 **Blast Statistics**http://www.biomedcentral.com/imedia/1870209046795595/supp1.xlsx Click here for file

Additional file 2 **Gene Ontology**http://www.biomedcentral.com/imedia/1490853420795595/supp2.xlsx Click here for file

Additional file 3 **Molecular Function Sequence Distribution.**http://www.biomedcentral.com/imedia/2017055384795595/supp3.xlsx Click here for file

Additional file 4 **Detailed Blast Result.xlsx**http://www.biomedcentral.com/imedia/1078330184795595/supp4.xlsx Click here for file

Additional file 5 **Additional Table KEGG Pathway Maps**http://www.biomedcentral.com/imedia/1441483719795595/supp5.xlsx Click here for file
